# Production of reproductively sterile fish by a non-transgenic gene silencing technology

**DOI:** 10.1038/srep15822

**Published:** 2015-10-29

**Authors:** Ten-Tsao Wong, Yonathan Zohar

**Affiliations:** 1 Department of Marine Biotechnology & Institute of Marine and Environmental Technology, University of Maryland Baltimore County, 701 E. Pratt Street, Baltimore, Maryland 21202, USA

## Abstract

We developed a novel bath-immersion technology to produce large numbers of infertile fish. As seafood consumption shifts from fishery harvests towards artificially propagated species, optimization of aquaculture practices will be necessary to maximize food production and minimize ecological impact. Farming infertile fish is the most effective genetic-containment strategy to support the development of environmentally-responsible aquaculture. We discovered that a molecular transporter, *Vivo*, can effectively carry the Morpholino oligomer (MO) across the chorion, enter the embryo and reach target cells. *Vivo*-conjugated MO against zebrafish *deadend* (*dnd*-MO-*Vivo*) effectively caused primordial germ cell mis-migration and differentiation into somatic cells, which resulted in generation of infertile fish. Optimal conditions were achieved when embryos, immediately after fertilization, were immersed with *dnd*-MO-*Vivo* at the initial concentration of either 60 or 40 μM followed by a lower serially diluted concentration. Under these conditions, 100% induced sterility was achieved even when the total immersion time was reduced from 24 to 5 hours. In 8 independent experiments, 736 adults developed from these conditions were all found to be infertile fish that possessed minimally-developed gonads that lacked any gametes. The results demonstrate that *dnd*-MO-*Vivo* bath immersion is an effective strategy to produce infertile fish without introducing transgenic modifications.

Aquaculture is progressively becoming more prevalent and vital to resolve the current and projected shortages in aquatic food availability. While the shift in reliance from fishery harvests to artificially propagated aquatic species continues, the increase in aquaculture activities poses a great threat to our ecosystem and environment. Non-native, selectively bred and, eventually, genetically modified farmed fish may escape from aquaculture containments, propagate and/or interbreed with wild stock, subsequently changing the genetic composition of populations or causing species extinction^1^,^2^. The use of reproductively sterile farmed fish will be the most effective strategy for genetic-containment, particularly in large scale operations, thereby achieving environmentally-responsible aquaculture practices. Infertile fish are desirable for a number of reasons. First, sterility carries environmental significance, as the escape of cultured sterile fish alleviates the threat to ecological balance and genetic contamination of wild populations^1^,^2^. Second, sterilization enhances muscle development by minimizing energy input toward gonadal growth and prevents sexual maturation that can cause deterioration of flesh quality and an increase in susceptibility to stress and disease^3^. Third, sterility is a means for producers to safeguard against unauthorized propagation of valuable fish strains.

In this study, we developed a bath immersion technology to produce infertile fish. We discovered that a molecular transporter comprised of a dendrimeric oligoguanidine with a triazine core, also known as *Vivo*^4^, can effectively traverse the Morpholino oligomer (MO) across the chorion and reach embryos. *Vivo*-conjugated MO against zebrafish *deadend* (*dnd-*MO-*Vivo*), an essential gene for zebrafish primordial germ cell (PGC) development^5^,^6^, effectively disrupted PGC development that led to the elimination of germ cells and resulted in the development of reproductive sterile fish. Our technology offers the aquaculture industry a convenient approach to maintain a fertile broodstock and generate infertile farmed fish without the introduction of transgenic modifications to foodfish.

## Results

### *Vivo*, a molecular transporter, effectively carries zebrafish *dnd*-MO across the chorion, transports it into the embryos and delivers it to target cells, resulting in disruption of PGC development

**Figure 1:**
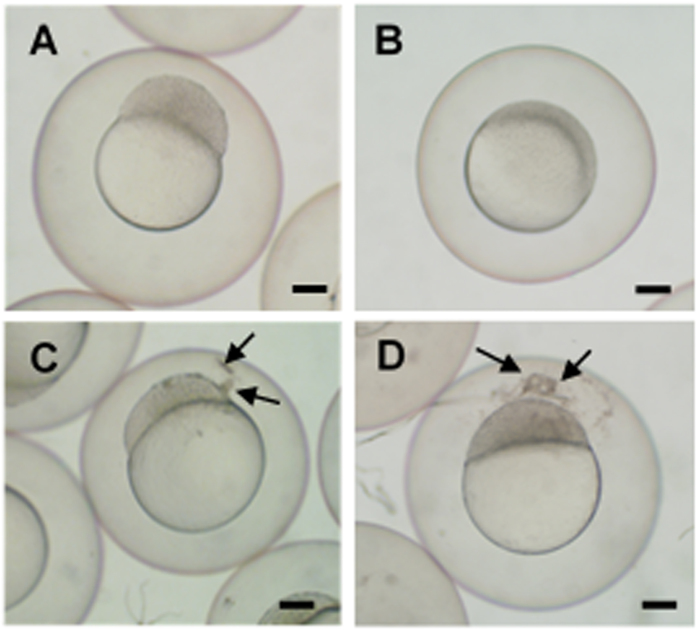
*Vivo* conjugated Morpholino oligomer (MO) caused developmental delays and uncharacterized aggregates in zebrafish embryos. When treated with 20 μM *dnd*-MO, embryos developed normally and reached (**A**) 1K cell stage after a 3 hour immersion and (**B**) 30-50% epiboly stage after a 5 hour immersion. When treated with 20 μM *Vivo* conjugated *dnd*-MO (*dnd*-MO-*Vivo*), embryo development was slightly delayed and only reached (**C**) 256-512 cell stage with aggregates found between the chorion and blastodisc (arrows) after a 3 hour immersion, and only reached (**D**) sphere stage with more aggregates (arrows) after a 5 hour immersion. Scale bar = 200 μm.

**Figure 2:**
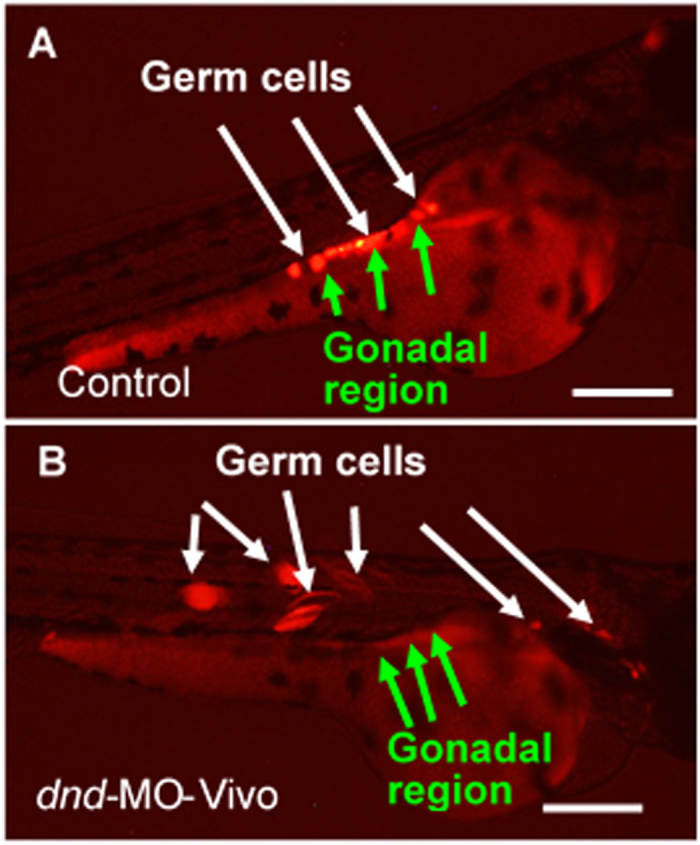
Zebrafish *dnd*-MO-*Vivo* disrupted germ cell development in zebrafish. (**A**) In control embryos immersed in only water or *dnd*-MO solution, germ cells migrated to the gonadal region and maintained their morphology as round-shaped cells. (**B**) Treatment of *dnd*-MO-*Vivo* caused germ cell mis-migration and eventually differentiation into other cell types that can be clearly seen by the change of their morphology. Scale bar = 200 μm.

Dnd has been proven to be an essential protein for germ cell development during embryogenesis in zebrafish^5^,^6^, loach^7^ and goldfish^8^. Knockdown of Dnd expression by microinjecting embryos with *dnd*-MO disrupted PGC development and resulted in reproductively sterile fish. These results indicate that blocking Dnd function is a viable approach to produce reproductively sterile fish. However, microinjection is not a technically or economically effective method for large-scale commercial operations. Hence, a bath immersion technology was developed to administer *dnd*-MO into embryos. In order to visualize and monitor germ cell development, *Tg (kop:DsRed-nanos3)* transgenic embryos that express DsRed specifically in the PGCs under the control of the germ-cell-specific *kop* promoter and *nanos3* 3′UTR^9^ were used to develop and optimize the bath immersion technology, utilizing zebrafish *dnd*-MO or a molecular transporter (*Vivo*) conjugated to *dnd*-MO (*dnd*-MO-*Vivo*). When embryos were immersed with 20 μM of *dnd*-MO, they developed normally and reached the 1K cell stage at 3 hours post-fertilization (hpf) and the 30–50% epiboly stage at 5 hpf (Fig. 1A, B) with no observable difference from the water-only controls. When embryos were immersed with 20 μM of *dnd*-MO-*Vivo*, their development was slightly delayed and only reached the 256–512 cell stage at 3 hpf (Fig. 1C) and the sphere stage at 5 hpf (Fig. 1D) with uncharacterized aggregates found around the inner part of the chorion or the surface of the blastodisc of embryos. These aggregates were not seen in the control groups that were immersed with water or 20 μM of *dnd*-MO (Fig. 1A,B). The results indicated that *Vivo* was able to act on and traverse the chorion, a thick acellular multi-layer coat, which resulted in the presence of the uncharacterized aggregates. In order to evaluate whether the *Vivo*-conjugated *dnd*-MO can enter embryos and eventually reach PGCs and knock down Dnd expression, the bath immersion protocol was optimized. A series titration in a 24-hour (total time) immersion bath was developed by starting the immersion at either 60, 40, 20 or 10 μM and ending at 5 μM of *dnd*-MO-*Vivo*. Under these immersion conditions, most embryos survived after treatment (Tables S1–4). The immersed embryos were examined using a fluorescence microscope at 2 to 3 days post-fertilization (dpf). In the control groups that were immersed in *dnd*-MO, control-MO-*Vivo* or water, PGCs migrated to the gonadal region and maintain their morphology as round-shaped cells (Fig. 2A). When embryos were initially treated with either 60 or 40 μM *dnd*-MO-*Vivo*, disruption of PGC development was found in all embryos examined, in which the PGCs were found to be at ectopic areas and some of them have differentiated into other cell types that can be clearly seen by the change of their morphology (Fig. 2B).

### Embryos immersed with *dnd*-MO-*vivo* developed into reproductively sterile adults

**Figure 3:**
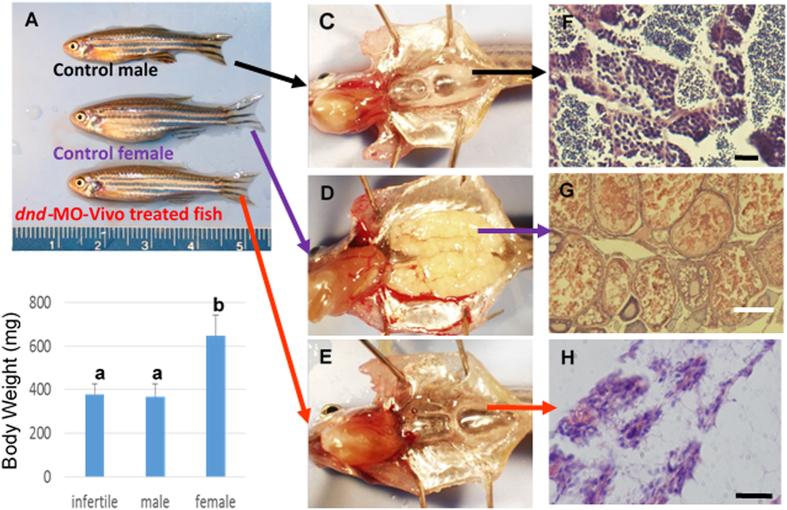
Zebrafish *dnd*-MO-*Vivo* induced sterility in zebrafish. Embryos initially treated with 60 or 40 μM of *dnd*-MO-*Vivo* developed into male-like adults. (**A**) No difference in appearance or overall size was observed between treated adult fish and control males. **(B**) No significant difference in body-weight (Mean ± SD) of 3-month-old fish (N = 12 by random sampling) was noted among *dnd*-MO-*Vivo* treated fish and control males (Data that share the same letter are not significantly different from each other). Examination of gonadal tissue showing (**C**) a fully-developed testis of a control male fish, (**D**) a fully-developed ovary of a control female fish, (**E**) the gonads of *dnd*-MO-*Vivo* treated fish that developed into a thin filament-like tissue. Photomicrographs (**F**–**H**) show (**F**) advanced spermatogenesis in the testis of a control male fish, (**G**) a well-developed ovary of a control female fish with oocytes at advanced stages of gametogenesis, (**H**) the gonad of *dnd*-MO-*Vivo* treated fish appears to be under-developed and surrounded with a large amount of adipocytes, without advanced gonadal structure or germ cells. Scale bar: white = 200 μm, black = 20 μm.

**Figure 4:**
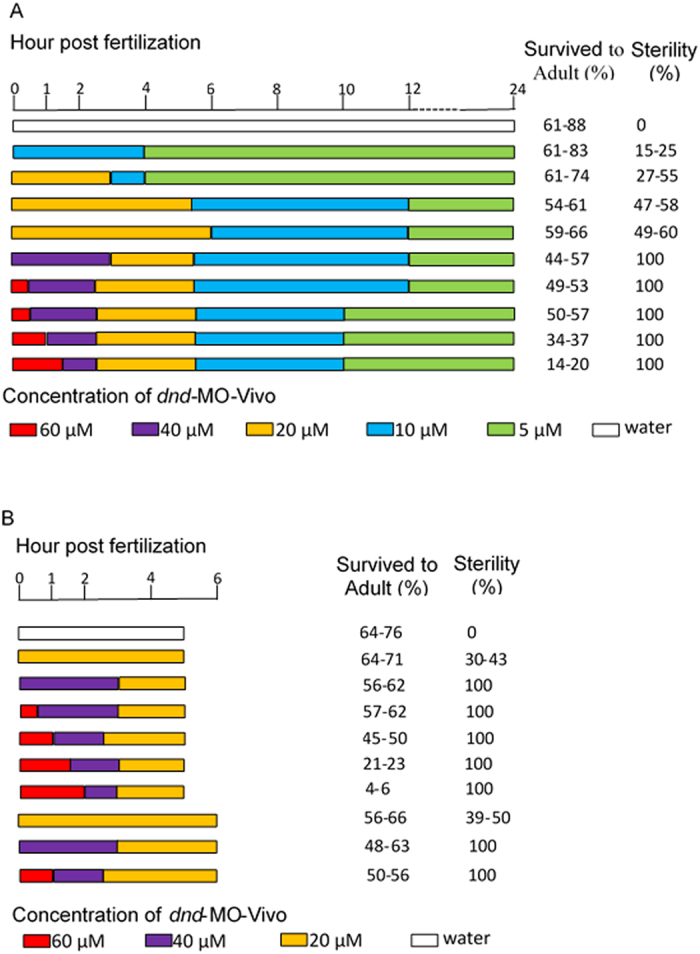
Zebrafish *dnd*-MO-*Vivo* treated embryos developed into infertile adults. In both (**A**) 24 hour immersions (data summarized from Tables S1–4) and (**B**) 5 to 6 hour immersions (data summarized from Tables S5–8), all the embryos that were initially immersed, immediately after fertilization, with 60 or 40 μM of zebrafish *dnd*-MO-*Vivo* developed into infertile fish. Data includes the water-only controls. Control-MO-*Vivo* and dnd-MO controls are presented in Table S1–8.

The bath-immersed embryos were raised to adults and the development of the fish was examined. A total of 1,201 adult fish were obtained from 4 separate 24-hour immersion experiments in which all of the 301 adult fish developed from embryos that were initially immersed with *dnd*-MO-*Vivo* at 60 μM for 0.5–1.5 hours, or at 40 μM for 3 hours, immediately after fertilization were found to be infertile. When embryos were initially immersed with 20 μM *dnd*-MO-*Vivo*, a total of 132 out of 255 fish were infertile. None of the control fish that were immersed with either *dnd*-MO, standard control MO-*Vivo* or water were found to be infertile (Table S1–4). The infertile adult fish have a male-like presence and exhibited no observable difference in survival rate, appearance (normality) and body weight from control males (Fig. 3A,B). Unlike control males, no sperm could be expressed from these male-like fish by gently pressing on their abdomen. Dissections were performed to examine gonad development in *dnd*-MO-*Vivo* immersed fish and control fish. The results revealed that testes (Fig. 3C) and ovary (Fig. 3D) development was normal in the control fish while gonad development was absent, except for a thin filament of connective tissue surrounded by fat tissue in all individuals that were initially treated with 60 or 40 μM *dnd*-MO-*Vivo* (Fig. 3E). Histological examination revealed that control male and female fish possessed fully formed gonads and active gametogenesis (Fig. 3F,G). In contrast, no gametogenesis was found in the filament-like gonad from *dnd*-MO-*Vivo* immersed fish (Fig. 3H). The treatment conditions and sterility results of 4 independent experiments that were immersed with *dnd*-MO-*Vivo* initially at either 60, 40, 20 or 10 μM, followed by a serially diluted concentration to final 5 μM for a total 24 hour immersion (Tables S1–4) are summarized in Fig. 4A.

### Treatment conditions affected survival rate and efficiency of sterility

The above results demonstrated that reproductively sterile fish can be obtained when fish embryos were initially immersed with either 60 or 40 μM *dnd*-MO-*Vivo* (Tables S2–4). However, 100% sterility could be achieved only when the embryos were immersed immediately after *in vitro* fertilization. If the naturally spawned embryos (about 30–60 minutes post-fertilization) were collected and used for immersion, only 52–68% of the embryos initially treated with 60 μM of *dnd*-MO-*Vivo* developed into infertile fish. Similar data were obtained when the immersion of *in vitro* fertilized embryos were started at 1 hpf, which resulted in only 44–59% of infertile fish (Table S4). These results indicated that treatment during the first hour after fertilization has an important effect on the efficiency of bath immersion. It also suggested that the total time of immersion could be shortened. As such, we optimized the protocol by reducing the length of bath immersion. In 4 separate 5 to 6-hour immersions, all 435 fish that were initially immersed with 60 or 40 μM of *dnd*-MO-*Vivo* developed into infertile adults. When embryos were immersed with 20 μM of *dnd*-MO-*Vivo*, only 30–50% of them developed into infertile adults (Tables S5–8). Furthermore, when embryos were immersed with 60 μM of *dnd*-MO-*Vivo* for longer than 1 hour the survival rate of treated individuals decreased dramatically. Only 4–6% of embryos survived to adults when they were initially treated with 60 μM of *dnd*-MO-*Vivo* for 2 hours (Table S6). When the total length of immersion was shortened to 4 hours, 100% sterility was not achieved even when embryos were initially treated with either 60 or 40 μM (data not shown). The treatment conditions and sterility results of 4 independent experiments that were immersed with *dnd*-MO-*Vivo* initially at either 60, 40 or 20 μM, followed by a serially diluted concentration for a total 5–6 hour immersion (Tables S5–8) are summarized in Fig. 4B. Optimal conditions resulting in 100% sterility induction were achieved when embryos, immediately after fertilization, were immersed with *dnd*-MO-*Vivo* at an initial concentration of either 60 or 40 μM.

## Discussion

To meet the demand of growing seafood consumption and compensate for the global decline of wild fishery stocks, it is imperative that highly efficient aquaculture practices are developed to enhance fishery production. However, the expansion of aquaculture operations also creates a great risk to our ecosystems and environment. Effective and practical fish sterilization technologies are crucial to resolve current and predicted threats posed by escapees from fish farms. Manipulating chromosome set normality by triploidization or interspecies hybridization is the most common method used to produce infertile fish^10^,^11^. However, some hybrids and triploids were found to be fertile^10^,^11^,^12^,^13^ and/or under-performing^14^,^15^,^16^. Another approach is the inhibition of gonadotropin-releasing hormone (Gnrh), a decapeptide required to develop and maintain a normal reproductive system in vertebrates, by transgenically expressing Gnrh antisense RNA to block Gnrh expression. Yet, low levels of Gnrh expression or development persist in some fish, resulting in a failure to completely induce sterility^17^. Recently, genetic germ-cell ablation by inducing germ cell death^18^,^19^,^20^ or disruption of germ cell development^9^ has been successfully developed to generate sterile fish^21^. The downside of this approach is that it requires the generation of transgenic fish in each species, thus introducing genetic modification into the fish and leading to long delays for regulatory approval as well as potential consumer resistance^22^,^23^. To overcome this hurdle, non-transgenic approaches to produce infertile fish are urgently needed to ease food safety concerns and propel aquaculture operations toward more environmentally responsible practices.

In this study, we developed a non-transgenic bath immersion method to induce fish sterility. Our technology is based on the administration of an antisense MO to block Dnd synthesis, which has been shown to be effective for inducing sterility in several fish species. To date, all the successful approaches were through the microinjection of *dnd*-MO into early-stage embryos^5^,^6^,^7^,^8^, which is not practical in commercial aquaculture. Immersion treatment can be used to administer bio-active or beneficial compounds to fish eggs or early-stage embryos. However, due to limited permeability of the chorion^24^, larger molecular compounds are not able to traverse the chorion and reach the embryo. Our innovation is based on the use of a molecular transporter that is able to effectively carry the compounds conjugated to it across the chorion, a thick acellular multi-layer envelope^24^,^25^, transport it into the egg or embryo, and deliver it to the target cells. This molecular transporter, also known as *Vivo*, was originally designed to induce endocytosis by transporting conjugated molecules into cells^4^. Similarly, after crossing the chorion, *Vivo* also promotes up-take of conjugated molecules into embryonic cells through endocytosis. Although it is not known how *Vivo*-conjugated compounds cross the chorion, and in particular whether they traverse through miniscule channels and pores, the aggregates found around the inner part of the chorion after the embryos were immersed with *dnd*-MO-*Vivo* and the ultimate disruption of PGC development indicated that *Vivo* is able to act on the chorion and transport the conjugated compound (*dnd*-MO-*Vivo*) across the barrier, thereby reaching the PGCs. These results were not seen in the control group of embryos that were immersed with *dnd*-MO. Our findings also indicated that Dnd protein may play an essential role in blocking PGCs from differentiating to other cell types, thus maintaining their germ cell characteristics. Knockdown of Dnd caused the differentiation of PGCs to fiber-like and other somatic cell types. This kind of cell morphology change and differentiation were not seen in other mis-migrated PGCs that were caused by over-expression of leukemia inhibitory factor^26^ or stromal-derived factor 1a^9^.

The identification of novel functionality of *Vivo* might also provide a method to efficiently deliver other larger molecules such as proteins, antibodies, DNAs and RNAs into fish eggs by conjugating them to *Vivo*, which has never been achieved without using micro-injection. Our results demonstrated that effective uptake of *dnd*-MO-*Vivo* and 100% sterility can be achieved under optimal treatment conditions. However, they also indicated that higher concentration and longer duration of immersion could increase mortality. When 100% sterility was achieved under optimized conditions, there were about 15–20% less fish that survived to adulthood (compared to control groups). Our results have shown that a slight delay of embryonic development occurred when embryos were immersed with *dnd*-MO-*Vivo*. In high concentration and longer duration of *dnd*-MO-*Vivo* immersion, the continuously high endocytosis activity during earlier embryonic stages may have slowed down and, eventually, irreversibly disrupted embryonic development, which led to the death of the embryos. Because these deaths happen at very early stages of development, it causes less destruction to aquaculture operations than deaths occurring at later stages of development. That being said, the complete pharmacology and animal/environmental toxicology of *Vivo*-MO will need to be studied before this technology is utilized by the aquaculture industry to produce sterile foodfish. It is also worth emphasizing that immersion delivery needs to be introduced shortly after fertilization. The delay of the initiation of immersion has been shown to reduce the efficiency of sterility induction. After fish eggs are fertilized and water-activated, the chorion permeability continues to decrease due to water hardening^24^,^27^,^28^, which may affect the up-take of *dnd*-MO-*Vivo*. As such, the first several hours after fertilization are the critical window for bath immersion effectiveness. Additionally, when the MO is taken up earlier, it may be distributed more efficiently to target cells and begin its knockdown function earlier, which also contributes to its effectiveness. MO antisense technology provides a promising approach to knock down gene expression and study gene function *in vivo*. However, due to unexpected non-specific interactions of MO with proteins and non-target mRNA^29^, and given that the addition of *Vivo* that may cause other non-specific effects, it is imperative that each species-specific *dnd*-MO-*Vivo* be evaluated carefully with the necessary controls.

Zebrafish were selected for initial development of the technical methodology, due to their short generation time and the large numbers of embryos that can be readily produced per pair (on a daily basis, if desired)^30^,^31^. Additionally, the transparent embryos and the availability of the transgenic *Tg (kop:DsRed-nanos3)* zebrafish line^9^ enables the visual observation of the early germ cells. Dnd is highly conserved in fish and its essential role in PGC development has been demonstrated in multiple species^5^,^6^,^7^,^8^. Moreover, development of PGCs and gonads within the embryo is an evolutionarily conserved mechanism in fish^32^. As such, it is expected that the technology can be successfully applied to other fish including a wide variety of aquaculture species. The success of our approach potentially provides an inducible method to generate reproductively sterile fish for aquaculture production without introduction of any genetic modifications into the foodfish. Additionally, the use of bath immersion makes it convenient to maintain a fertile broodstock population by simply omitting the treatment of the embryos. Our technology can thus be used for genetic containment and cost-effective aquaculture operations, which will contribute to the development of environmentally and economically sustainable production to meet the growing global demand for seafood.

## Methods

### Animals and Ethics

Zebrafish were maintained and staged as previously described^33^. All of the experimental procedures and protocols described in this study were approved by the University of Maryland Animal Care and Use Committee and adhered to the National Research Council’s Guide for Care and Use of Laboratory Animals.

### Bath immersion

To visualize PGC development, embryos of *Tg (kop:DsRed-nanos3)* zebrafish that carry maternal DsRed-labeled PGCs were generated by *in vitro* fertilization and 50 eggs in duplication were set aside for fertilization rate measurement when embryos developed to the 16 to 32 cell stage. To perform *in vitro* fertilization, sperm was collected from 4–8 males and suspended in 0.1 ml of ice-cold Hank’s solution, and eggs from 4–8 females were collected in a 10 cm Petri dish following a published protocol^33^. 30–50 μl of the sperm/Hank’s solution was added to the eggs and mixed gently, followed by the addition of 2 ml of fresh tank system water for 1 minute. An additional 10 ml of fresh tank system water was added to the Petri dish for another 2-minute incubation and then the fertilization solution was replaced with15–20 ml of fresh tank system water. 50–80 eggs were transferred into each well of 48-well plates that contained 300 μl of fresh tank system water. After the allocation of eggs, system water in each well was replaced with 200–300 μl of water that initially contained 0–60 μM of zebrafish *dnd*-MO (5′-GCTGGGCATCCATGTCTCCGACCAT-3), *dnd*-MO-*Vivo* or standard control MO-*Vivo* (5′-CCTCTTACCTCAGTTACAATTTATA-3′) (Gene Tools LLC. Philomath, OR, USA). After 0.5–3 hours of incubation, the initial concentration of *dnd*-MO, *dnd*-MO-*Vivo* or control-MO-*Vivo* was gradually decreased by adding 1-fold of water each time to the desired final incubation concentration. To prevent rapid change of the *dnd*-MO-*Vivo* concentration during the dilution, one half of the immersion solution of each treatment was transferred and mixed with 1 volume of water and then the solution was gradually transferred back to each well. The total immersion time was 4 to 24 hours, depending on each immersion condition. After the treatment, the immersion solution was gradually replaced with fresh water and embryos were transferred to 10 cm Petri dishes and incubated in a 28–29 °C incubator. At 2–3 dpf, embryos were examined using a MZ12 stereomicroscope (Leica, Buffalo Grove, USA), or an Axioplan2 fluorescence microscope (ZEISS, Thornwood, NY, USA). Both microscopes were equipped with a DP70 digital camera (Olympus, Center Valley, PA, USA). All the embryos were raised to adults to determine their fertility.

### Histology

Zebrafish were euthanized in 0.016% tricaine (ethyl-3-aminobenzoate methanesulfonic acid; Sigma-Aldrich) solution in water, and gonads were removed and fixed with 4% paraformaldehyde in phosphate buffered saline (PBS) at 4 °C overnight. After two rinses in PBS, the fixed gonads were processed through successive ethanol treatments (50%, 70%, 95%, and 100%), followed by two xylene treatments, and embedded in paraffin. The serial 5 μm paraffin sections were prepared using a HM340 microtome (Leica). For histology, the sections were stained with hematoxylin-eosin and examined via light microscopy.

### Statistical Analysis

Data are presented as the mean and standard deviation. For statistical analysis one-way ANOVA was applied followed by a Bonferroni–Dunn test using the SAS program. Significance was accepted at *p* < 0.05.

## Additional Information

**How to cite this article**: Wong, T.-T. and Zohar, Y. Production of reproductively sterile fish by a non-transgenic gene silencing technology. *Sci. Rep.* **5**, 15822; doi: 10.1038/srep15822 (2015).

## Supplementary Material

Supplementary Tables
