# Supplementary material for: Production of reproductively sterile fish by a non-transgenic gene silencing technology
Source: Sci Rep. 2015 Oct 29;5:15822. doi: 10.1038/srep15822 (PMC4625178; doi:10.1038/srep15822)
Supplement: Supplementary Tables [file srep15822-s1.pdf]

Supplementary information

Title: Production of reproductively sterile fish by a non-transgenic gene silencing technology

Authors and Authors' affiliation:

Ten-Tsao Wong and Yonathan Zohar

Department of Marine Biotechnology & Institute of Marine and Environmental Technology

University of Maryland Baltimore County

701 E. Pratt Street

Baltimore, Maryland 21202, USA

Supplementary Tables S1-8. Results of *dnd*-MO-Vivo, *dnd*-MO or control MO-Vivo bath immersion using *in vitro* fertilized *T*g *(kop:DsRed-nanos3)* transgenic zebrafish embryos.

Table S1. 24 hr immersion

50 embryos per group in triplicates, 89% fertilization rate
A: *dnd*-MO-Vivo 20 µM for 3 hrs, 10 µM for 1 hr and 5 µM for 20 hrs
B: *dnd*-MO-Vivo 10 µM for 4 hrs and 5 µM for 20 hrs
C: Water-only solution as control

	A1	A2	A3	B1	B2	B3	C1	C2	C3
embryos hatched (2dpf)	37	41	38	43	40	37	41	43	39
and hatching rate	83%	92%	85%	96%	89%	83%	92%	96%	87%
adult fish obtained	30	27	33	37	32	27	33	39	31
number of males	5	7	3	19	9	10	13	14	10
number of females	16	10	12	11	15	13	20	25	21
number of infertile fish	9	10	18	7	8	4	0	0	0
% of infertile fish	30%	27%	55%	19%	25%	15%	0%	0%	0%
Average % of infertile fish		37%			20%			0%	

Table S2. 24 hr immersion

60 eggs per group in duplicates, 91% fertilization rate

A: *dnd*-MO-Vivo 60 µM for 0.5 hr, 40 µM for 2 hrs, 20 µM for 3 hrs, 10 µM for 6.5 hrs and 5 µM for 12 hrs

B: *dnd*-MO-Vivo 40 µM for 3 hrs, 20 µM for 2.5 hrs, 10 µM for 6.5 hrs and 5 µM for 12 hrs

C: *dnd*-MO 60 µM for 0.5 hr, 40 µM for 2 hrs, 20 µM for 3 hrs, 10 µM for 6.5 hrs and 5 µM for 12 hrs

D: Control MO-Vivo 60 µM for 0.5 hr, 40 µM for 2 hrs, 20 µM for 3 hrs, 10 µM for 6.5 hrs and 5 µM for 12 hrs

E: Water-only solution as control

	A1	A2	B1	B2	C1	C2	D1	D2	E1	E2
embryos hatched (2 dpf)	43	45	42	45	51	53	37	45	53	51
and hatching rate	78%	82%	76%	82%	93%	97%	67%	82%	97%	93%
adult fish obtained	27	29	26	31	38	32	29	26	40	34
number of males	0	0	0	0	16	19	10	19	27	20
number of females	0	0	0	0	22	13	19	7	13	14
number of infertile fish	27	29	26	31	0	0	0	0	0	0
% of infertile fish	100%	100%	100%	100%	0%	0%	0%	0%	0%	0%
Average % of infertile fish		100%		100%		0%		0%		0%

Table S3. 24 hr immersion

80 embryos per group in duplicates, 92% fertilization rate

A: *dnd*-MO-Vivo 40 µM for 3 hrs, 20 µM for 2.5 hrs, 10 µM for 6.5 hrs and 5 µM for 12 hrs

B: *dnd*-MO-Vivo 20 µM for 5.5 hrs, 10 µM for 6.5 hrs and 5 µM for 12 hrs

C: *dnd*-MO-Vivo 20 µM for 6 hrs, 10 µM for 6 hrs and 5 µM for 12 hrs

D: Water-only solution as control

	A1	A2	B1	B2	C1	C2	D1	D2
embryos hatched (2dpf)	57	54	66	62	63	56	71	68
and hatching rate	77%	73%	89%	84%	85%	76%	96%	92%
adult fish obtained	37	32	40	45	36	44	48	54
number of males	0	0	11	17	10	11	23	21
number of females	0	0	6	7	4	4	25	33
number of infertile fish	37	32	23	21	22	29	0	0
% of infertile fish	100%	100%	58%	47%	59%	66%	0%	0%
Average % of infertile fish		100%		53%		63%		0%

Table S4. 24 hr immersion

60 embryos per group in duplicates, 94% fertilization rate.
A: *dnd*-MO-Vivo 60 µM for 0.5 hr, 40 µM for 2 hrs, 20 µM for 3 hrs, 10 µM for 4.5 hrs and 5 µM for 14 hrs
B: *dnd*-MO-Vivo 60 µM for 1 hr, 40 µM for 1.5 hrs, 20 µM for 3 hrs, 10 µM for 4.5 hrs and 5 µM for 14 hrs
C: *dnd*-MO-Vivo 60 µM for 1.5 hrs, 40 µM for 1 hr, 20 µM for 3 hrs, 10 µM for 4.5 hrs and 5 µM for 14 hrs
D: Water-only solution as control
E*: *dnd*-MO-Vivo 60 µM for 0.5 hr, 40 µM for 2 hrs, 20 µM for 3 hrs, 10 µM for 4.5 hrs and 5 µM for 14 hrs

	A1	A2	B1	B2	C1	C2	D1	D2	E1	E2
embryos hatched (2dpf)	41	40	33	30	26	22	52	50	45	47
and hatching rate	72%	72%	58%	53%	46%	39%	92%	88%	84%	83%
adult fish obtained	28	32	19	21	11	8	39	43	34	29
number of males	0	0	0	0	0	0	16	15	12	9
number of females	0	0	0	0	0	0	23	28	7	3
number of infertile fish	28	32	19	21	11	8	0	0	15	17
% of infertile fish	100%	100%	100%	100%	100%	100%	0%	0%	44%	59%
Average % of infertile fish		100%		100%		100%		0%		52%

*Started the treatment at 1 hr post-fertilization.

Table S5. 6 hr immersion

60 embryos per group in triplicates, 93% fertilization rate

A: *dnd*-MO-Vivo 60 µM for 1 hr, 40 µM for 1.5 hrs and 20 µM for 3.5 hrs

B: *dnd*-MO-Vivo 40 µM for 3 hrs and 20 µM for 3 hrs

C: *dnd*-MO-Vivo 20 µM for 6 hrs

D: control MO-Vivo solution 60 µM for 1 hr, 40 µM for 1.5 hrs and 20 µM for 3.5 hrs

	A1	A2	A3	B1	B2	B3	C1	C2	C3	D1	D2	D3
embryos hatched (2dpf)	35	42	34	45	47	40	52	43	48	32	40	37
and hatching rate	62%	75%	60%	80%	84%	71%	93%	77%	86%	57%	71%	66%
adult fish obtained	28	31	29	35	33	27	37	32	31	25	34	31
number of males	0	0	0	0	0	0	14	11	13	17	16	10
number of females	0	0	0	0	0	0	8	5	6	8	18	21
number of infertile fish	28	31	29	35	33	27	15	16	12	0	0	0
% of infertile fish	100%	100%	100%	100%	100%	100%	41%	50%	39%	0%	0%	0%
Average % of infertile fish		100%			100%			43%			0%	

Table S6. 5 hr immersion

60 embryos per group in duplicates, 82% fertilization rate
A: *dnd*-MO-Vivo 60 µM for 1 hr, 40 µM for 2 hrs, 20 µM for 2 hrs
B: *dnd*-MO-Vivo 60 µM for 1.5 hrs, 40 µM for 1.5 hrs, 20 µM for 2 hrs
C: *dnd*-MO-Vivo 60 µM for 2 hrs, 40 µM for 1 hr, 20 µM for 2 hrs
D: Water-only solution as control

	A1	A2	B1	B2	C1	C2	D1	D2
embryos hatched (2 dpf)	33	35	21	17	14	13	49	45
and hatching rate	67%	71%	42%	34%	28%	26%	99%	91%
adult fish obtained	23	26	11	12	2	3	36	39
number of males	0	0	0	0	0	0	16	22
number of females	0	0	0	0	0	0	20	17
number of infertile fish	23	26	11	12	2	3	0	0
% of infertile fish	100%	100%	100%	100%	100%	100%	0%	0%
Average % of infertile fish		100%		100%		100%		0%

Table S7. 5 hr immersion

60 embryos per group in duplicates, 86% fertilization rate

A: *dnd*-MO-Vivo 60 µM for 1 hr, 40 µM for 1.5 hrs and 20 µM for 2.5 hrs

B: *dnd*-MO-Vivo 40 µM for 3 hrs and 20 µM for 2 hrs

C: *dnd*-MO-Vivo 20 µM for 5 hrs

D: Water-only solution as control

	A1	A2	B1	B2	C1	C2	D1	D2
embryos hatched (2 dpf)	32	30	38	36	43	41	45	43
and hatching rate	62%	58%	73%	69%	83%	79%	87%	83%
adult fish obtained	26	23	32	29	35	33	37	33
number of males	0	0	0	0	13	15	24	19
number of females	0	0	0	0	7	8	13	14
number of infertile fish	26	23	32	29	15	10	0	0
% of infertile fish	100%	100%	100%	100%	43%	30%	0%	0%
Average % of infertile fish		100%		100%		37%		0%

Table S8. 5 hr immersion

60 embryos per group in duplicates, 91% fertilization rate

A: *dnd*-MO-Vivo 60 µM for 0.5 hr, 40 µM for 2.5 hrs and 20 µM for 2 hrs

B: *dnd*-MO-Vivo 20 µM for 5 hrs

C: Water-only solution as control

	A1	A2	B1	B2	C1	C2
embryos hatched (2 dpf)	38	42	50	46	54	51
and hatching rate	69%	76%	91%	84%	98%	93%
adult fish obtained	31	34	36	39	40	38
number of males	0	0	16	17	26	16
number of females	0	0	7	5	14	22
number of infertile fish	31	34	13	17	0	0
% of infertile fish	100%	100%	36%	43%	0%	0%
Average % of infertile fish		100%		40%		0%
